# Integrating the latest biological advances in the key steps of a food packaging life cycle

**DOI:** 10.3389/fnut.2023.1223638

**Published:** 2023-07-27

**Authors:** Aynura Rzayeva, Fanny Coffigniez, Nizami Zeynalov, Nathalie Gontard, Valérie Guillard

**Affiliations:** ^1^IATE, Agro Polymers Engineering & Emerging Technology, Univ Montpellier, INRAE, Institut Agro, Montpellier & CIRAD, Montpellier, France; ^2^Nanostructured Metal-Polymer Catalysts, Institute of Catalysis and Inorganic Chemistry, Ministry of Science and Education Republic of Azerbaijan, Baku, Azerbaijan

**Keywords:** sustainability, biodegradable, active food packaging, natural active components, usage benefit, post-usage fate

## Abstract

This literature review provides a focus on the potential of integrating the latest scientific and technological advances in the biological field to improve the status of the key steps of a food packaging life cycle: production, usage, post-usage, and long-term fate. A case study of such multi-biological food packaging is demonstrated based on the use of PHAs (polyhydroxyalkanoates) polymer, a microbiologically produced polymer from non-food renewable resources, activated by the use of bioactive components to enhance its usage benefits by reducing food loss and waste, displaying potential for reusability, compostability as post-usage, and finally, being ultimately biodegradable in most common natural conditions to considerably reduce the negative impact that persistent plastics have on the environment. We discuss how designing safe and efficient multi “bio” food packaging implies finding a compromise between sometimes contradictory functional properties. For example, active antimicrobials help preserve food but can hamper the ultimate biodegradation rate of the polymer. This review presents such antagonisms as well as techniques (e.g., coatings, nanoencapsulation) and tools (e.g., release kinetic) that can help design optimized, safe, and efficient active food packaging.

## 1. Introduction

**Figure 1:**
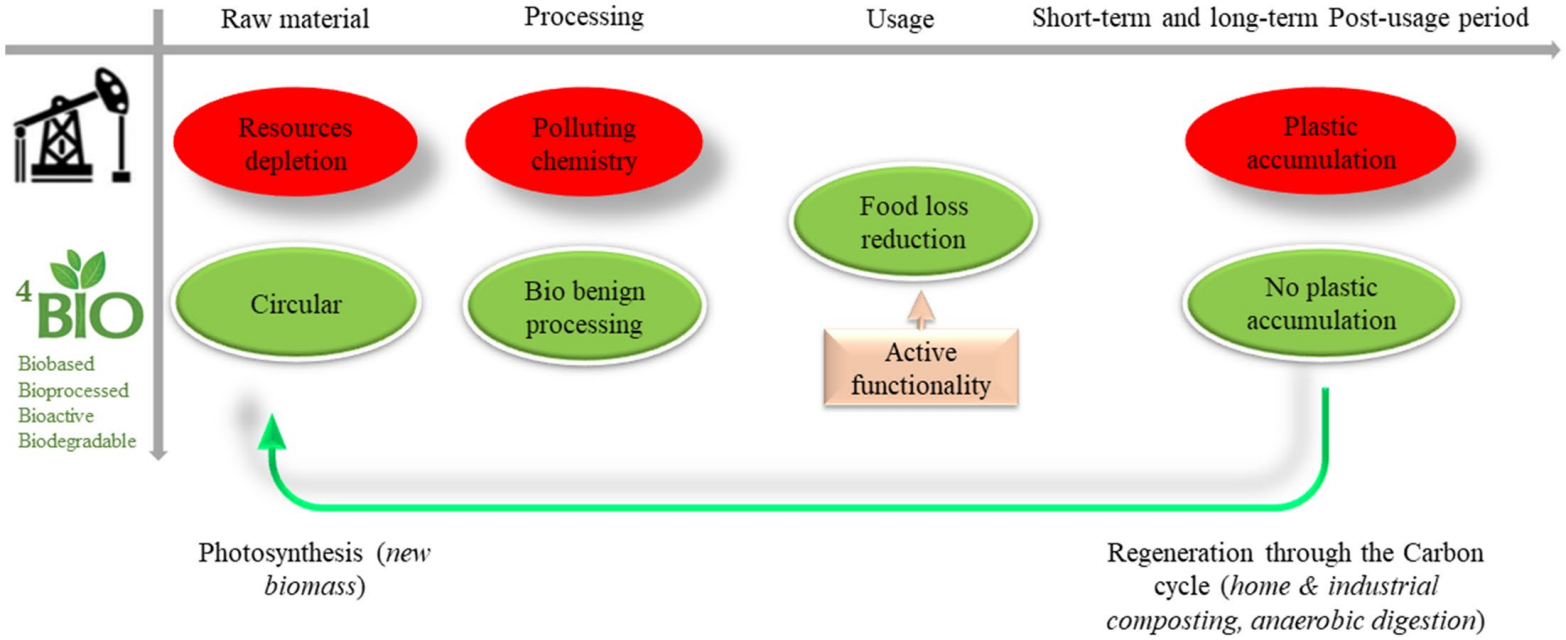
The expected effect of substituting fossil-based plastic materials with BIO^4^ ones – in the life cycle of food packaging materials (BIO^4^ = biobased, bioprocessed, bioactive, and biodegradable).

Although packaging plays a crucial role in the food supply chain by maintaining the quality and safety of food for a certain period, environmental problems due to resource depletion and persistent plastic accumulation from food packaging are of increasing concern. Indeed, the world’s plastic production is expected to increase three-fold by 2060, and ⅓ of this production will be used for packaging development (1, 2). In the meantime, 90% of this plastic ends up in the environment (through littering, landfilling, mismanagement, etc.): 3% directly in the ocean and 87% on the terrestrial continent (in soil), where they are fragmented through physical abrasion into micro and nanoparticles that diffuse into all environmental compartments (air, soil, water, and living organisms), resulting in considerable environmental and biological damage (1, 3). In this context, the emergence of new materials allowing the overall packaging sustainability to be increased is paramount: it clearly means (1) minimizing overall resource use and process step impacts, (2) ensuring product preservation to reduce food loss and waste, (3) reducing the burden of post-usage plastic waste management (e.g., by being compostable or recyclable) and, (4) paying special attention to the long-term outcome that can lead to the accumulation of plastic in the environment in the final stage of the materials life chain (3–5). One solution to address these multiple issues is to focus on materials that provide a biological solution at each stage: a biological resource, bioprocess transformation, bioactivity to better preserve food, and finally biological digestibility in the post-usage stages (Figure 1).

Therefore, to correctly use *biobased* resources for packaging development (e.g., plant or microbial polymers), it is necessary to use materials that are compatible with biomass temporal availability and the regeneration time and to avoid competition with food and feed uses. Moreover, *bioprocess* transformation refers to the use of biotechnologies to synthesize the material (e.g., microbial cells) with the net advantage that the range of resources to be used is considerably enlarged. For instance, with biotechnologies, it is possible to use organic wastes and other residues (6, 7). *Bioprocesses* also appear to be good alternatives to conventional chemical synthesis, because they require less time and additives, which can result in health problems in living marine and terrestrial organisms (endocrine disruptors, etc.) (8–10). *Bioactive* packaging refers to the integration of active bio components in the polymer, such as natural antimicrobials or antioxidants which can increase the packaging usage benefit by preserving food from deterioration, which leads to food loss and waste (FLW) mitigation, as demonstrated by several authors (11–13). Finally, *biodegradable packaging* means that the material could be disintegrated and then assimilated by microorganisms and digested into small unitary molecules (H_2_O, CO_2_, CH_4_) to produce a new microbial biomass. Ultimate biodegradation is currently the only solution to reduce conventional plastic accumulation in the environment (3).

Although several articles have already highlighted the importance of biodegradable packaging [see among others (14)], or the relevance of active, and smart biodegradable packaging for the food sector (15), or the applicability of biobased packaging for long-shelf-life foods (16), no publications to date have addressed all the four ‘bio’ aspects in a holistic approach to the packaging sustainability. It should be noted that in the web of sciences with the keywords “biodegradable, bioactive packaging” AND “biodegradable, bioactive, biobased packaging” AND “biodegradable, antimicrobial packaging” AND “biodegradable, biobased, antimicrobial packaging,” we obtained 90, 5, 215, and 6 review articles, respectively (carried out in February 2023).

In this context, the aim of this paper is to discuss the relevance of developing active packaging materials combining the four “bio” aspects (biobased, bioprocessed, bioactive, and biodegradable) by highlighting their challenges and limitations. Examples will be provided from commercial products or polymers that are still in development (e.g., PHAs, a family of polymers that, promisingly, covers the four “bio” aspects). This review highlights the importance of a reasoned-integrated approach to develop bio-benign, biodegradable, efficient active food packaging starting from the food needs in terms of preservation without compromising the ultimate biodegradability of the material. The importance of the use of biobased, bioprocessed, and biodegradable material will be discussed first, with examples of different polymers more or less in accordance with these aspects. The importance of the strategy of bioactive molecule incorporation is then developed, with the presentation of cutting-edge ways for incorporating active ingredients into biopolymers. Finally, the post-usage fate of the bioactive, biodegradable packaging materials is discussed.

## 2. The importance of using biobased, bioprocessed, and biodegradable materials for food packaging

### 2.1. Definition of biobased, bioprocessed, and biodegradable materials

#### 2.1.1. Biobased

The fear of a near future where oil-based resources are lacking and the societal demand for more ‘green’ materials have led to the development of polymers derived from renewable biological resources (‘biobased’).

For this purpose, research efforts have long focused on transforming plant or other non-fossil biogenic feedstocks into materials identical (e.g., bio-PET, bio-PE) or close to those derived from petroleum (e.g., PLA). At the same time, efforts have been made regarding the development of biodegradable solutions to solve the issue of plastic persistence in the environment, leading to the emergence, in the early 1990s, of the ‘bioplastics’ market, which includes both biodegradable and biobased plastics, with unfortunately a high degree of confusion regarding the distinction between them (17, 18). “Biobased” means materials partially or fully produced from biomass resources (such as sugar cane, hydrolyzed maize, rice or potato starch, etc.), irrespective of the end-of-life fate of the material (17). As such, a biobased polymer may be not biodegradable, such as bio-polyethylene (bio-PE), which is not more biodegradable than its oil-based counterpart. Several publications have sought to clarify this terminology and denounce the variations coming from the overall misinterpretation of the ‘bio’ prefix (17–19).

#### 2.1.2. Bioprocessed

Two categories of biobased polymers can be considered (20): the first one consists of materials whereby the chemical structure of the biomass feedstock is not maintained. This is the case, for instance, when biomass is used to obtain soluble sugars, which are then fermented to produce monomers for the polymerization (e.g., lactic acid to produce poly(lactic acid) or ethylene to produce biobased polyethylene). This is the route followed to obtain polymers that are identical to those derived from petroleum (e.g., bio-PET, bio-PE). The second category corresponds to a group of polymers directly synthetized by living organisms (plants, algae, microorganisms, etc.) for which the initial chemical structure is preserved after extraction and purification and then used as such [e.g., polyhydroxyalkanoates (21)], polymalic acid (22) or only slightly modified [e.g., celluloses acetates (23)].

To manufacture compounds belonging to the first category, significant efforts must be placed on the plant transformation, with selective chemical processes required to remove chemical functionality afforded naturally by many biobased feedstocks (e.g., many natural molecules contain oxygen or nitrogen, while this is not the case for fossil-based hydrocarbons). Agricultural crops produce precursors: mostly starch and soluble sugars, that can be directly used to obtain biobased polymers, or building blocks derived from selective transformation used to extract, fractionate, and deconstruct plant cell-wall polymers (cellulose, hemicellulose, pectin, lignin, etc.) (24). Additional chemical transformations are often needed to obtain the necessary functionality for polymerization of the biobased building blocks into polymers.

Beyond the economic and environmental cost of all this necessary chemistry, the use of dedicated agricultural crops for producing biobased polymers is highly controversial in a period of fears for worldwide food security (25). Most lifecycle analyses (LCA) show that biobased polymers are better than their oil-based equivalents in aspects such as GHG emissions and fossil fuel consumption but not for other indicators such as eutrophication, land occupation, etc., which leaves the question of environmental superiority of biobased polymers for further debate (26, 27).

In light of the questionable economic viability and environmental issues of biobased polymers chemically synthesized from biobased building blocks, a reasonable guideline would be to focus on the second category of polymers, those directly synthetized by living organisms and used after extraction/purification with minimal chemical transformations. To not compete with the food chain, they should be produced from agricultural or food by-products or residues. The use of residues and even organic wastes as feedstock to produce polymers is gaining more and more attention, especially in line with the development of circular economy schemes (7). They provide significant environmental benefit, provided that their collection, transport, and transformation are regionally-reasoned and do not necessitate expensive pre-treatments and transportation (28, 29).

#### 2.1.3. Biodegradable

Biodegradability is the intrinsic property of a material to be fully degraded by living microorganisms (e.g., soil microorganisms) into a new biomass and small non-toxic molecules such as water, carbon dioxide (CO_2_), and/or methane (CH_4_) in widespread natural environmental conditions and in a reasonable timeframe compatible with human life cycles (17). The term biodegradable is often and misleadingly applied to plastics that are biobased but not necessarily biodegradable, or that undergo degradation (e.g., lowering of the molar masses of macromolecules that form the material) but without complete, ultimate mineralization (30). The biodegradability of a polymer depends only on its chemical structure and not the carbon source. Polymers that are compostable are not necessarily biodegradable under ambient environmental conditions. For instance, PLA needs to reach a minimal temperature of approximately 60°C to be biodegraded, which can be achieved in industrial composting plants but not in soil for instance or even domestic compost (19). The risk of considering a polymer that turns out not to be biodegradable under ambient environmental conditions is the production of persistent micro and nano plastics with long-term adverse effects for the entire biosphere (3).

In light of the above, *nonfood, nonfeed biobased,* and *minimally bioprocessed* polymers appear to be the best compromise of biobased and bioprocessed polymers. In addition, care must be taken regarding the type of biodegradation that the material can achieve: *ultimate biodegradation*, e.g., complete degradation into mineral molecules, under common ambient environmental conditions, and in a reasonable timeframe are paramount.

### 2.2. The various biobased and/or bioprocessed and/or biodegradable biopolymers

#### 2.2.1. Overview of the various types of biopolymers

Table 1 summarizes the various types of biopolymers already marketed and commercially available and also those that are still in development. Clearly, currently marketed biopolymers are not fully “bio” from their resources nor their end-of-life, or present limited usage benefit. They are either derived from food resources (e.g., PLA, PBSA, starch-based blends, or commercial PHBV) or not fully biodegradable under natural conditions (e.g., PLA), or not water resistant (e.g., starch-based blends).

**Table 1:** The various biopolymers in development or available on the market.^1^

Biopolymer	Global annual production in kilotons/year (2022 data)^1^	TRL achieved	Resources: biobased (origin of material)	Usage benefit: advantages/disadvantages	Post usage fate
Bio-sourced, non-biodegradable, and non-compostable biopolymers ➔ 1-bio					
Bio-PET	93	−⑨−	Commonly 100% of biobased ethylene glycol (EG) and petroleum-derived terephthalic acid (TA); 100% biobased EG, and PTA production is feasible	Very good processability, versatility, O_2_/CO_2_/H_2_0 barrier	Recyclable, non-biodegradable
Bio-PA	246	−⑨−	100% (vegetable biomass)	Very good processability, versatility, O_2_/CO_2_ barrier	Recyclable, non-biodegradable
Bio-PE	329	−⑨−	100% (e.g., sugar cane, sugar beet)	Very good processability, extensibility, versatility, H_2_0 barrier	Recyclable, non-biodegradable
Oil-based, biodegradable, and home-compostable biopolymers ➔ 1-bio					
PBAT	100	−⑨−	Fossil	Properties similar to LDPE, flexible (strain at break ∼710%) and tough, PBAT is used primarily in combination with another polymer	Home compostable
PBSA	20	−⑨−	20–100% (corn, cane sugar, cassava, beets)	Sensitivity to humidity, temperature variations, and UV	Home compostable
Bio-sourced and compostable (industrial conditions) bio-polymers, conventional chemistry for polymerization ➔ 2-bio					
PLA	460	−⑨−	100% (corn, cane sugar, cassava, beets)	Low- and high-temperature behavior to be improved	Industrially compostable EN 13432 or NFT 51–800
Biobased, biodegradable (in natural conditions), home-compostable, biosynthesized biopolymers ➔ 3-bio					
Cellulose	273.31 million^2^	−⑨−	> 90% (cellulose pulp)	Tear resistance at low temperatures, lack of barriers (oxygen, fat, etc.)	Home compostable
Starch-based materials	397	−⑨−	30–100% (maize, wheat, potato) Competition w/food	Sensitivity to temperature variations, not water resistant	Industrially compostable EN 13432 or NFT 51–800 & Home compostable
PHB/coHV (from noble food resources)	50	−⑨−	100% (corn, sugars, beet)	Barrier properties similar to that of PP, brittle if low HV	Home compostable
PHB/coHV (from organic wastes and residues)	Still pilot-scale production	−⑥−	100% (agricultural and food residues and wastes, urban wastes)	Barrier properties similar to that of PP, brittle if low HV	Home compostable
PHBH	Undisclosed	−⑨−	100% (pure oils, currently)	n/a	Home compostable
mcl-PHA^3^	Still experimental production	−③−	100% (pure food resources, currently)	n/a	Home compostable

^1^http://www.european-bioplastics.org/bioplastics/materials/. ^2^*Global annual production*: Paper and cardboard for packaging: « La demande mondiale des matériaux des emballages papier devrait atteindre 273,31 millions de tonnes en 2022 contre 195,72 millions en 2014 » (https://www.graphiline.com/article/21772/previsions-marche-mondial-emballages-papier). ^3^Medium-chain-length PHA.

Only cellulose-based materials and PHAs obtained from non-food resources (such as PHBV) appear to fulfill all the criteria.

#### 2.2.2. PHAs, an example of biobased, bioprocessed, and biodegradable polymers

PHAs comprise a family of polymers with very versatile properties (water-insoluble, hydrophobic, thermoplastic). They can be molded into rigid or semi-rigid items using thermomechanical processes, and in their final shape, they exhibit good oxygen barrier properties that make them promising for fresh and other processed food packaging applications (31).

Commercial PHAs are currently nearly exclusively the copolymer polyhydroxy(butyrate-co-valerate), P(HB-co-HV). P(HB-co-HV) is a copolymer, meaning that it is a combination of two monomers, namely hydroxy-butyrate and hydroxy-valerate. P(HB-co-HV) is currently produced using pure microbial cultures fed with high-purity substrates that require (1) sterility (2) high energy, and (3) pure glucose or corn steep liquor as feedstock (48% of the total production cost). This contributes to a prohibitive market price (5 €/kg) to be used as a commodity polymer.

To address this issue, and to create a virtuous cycle rather than depleting one by using organic residues instead of apure carbon source, researchers have developed bioconversion processes of agri-food residues using optimized eco-efficient mixed microbial cultures (MMC). These processes decrease the investments and operating costs of the P(HB-co-HV) conversion with respect to pure culture and make it easier to use cheaper by-products such as feedstock (6, 32, 33). However, these processes are not yet available at an industrial scale.

Once produced, PHA macromolecules must be extracted and purified to remove all impurities (e.g., proteins, inorganic compounds) that can hamper its further use as a thermo-mechanical processable polymer. To do this, after harvesting cells from the bioreactor, flocculation, centrifugation, or filtration is used to remove the aqueous phase from the sedimented biomass. After a thermal drying or lyophilization step, the PHA-rich biomass is ready for extraction and purification (34, 35). Most people utilize methods on a laboratory scale such as solvent-based extraction (using halogenated solvents or halogen-free solvents such as butanol, or acetone, etc.), enzymatic (Alcalase^®^, lysozyme) or mechanical techniques (ultrasonication, high-pressure homogenization, osmotic pressure), or combinations of these methods. These methods result in cell disruption, enabling polymer solubilization in the solvent used. The last steps are then washing and drying the polymer to obtain a powder that can be stored and subsequently used (36).

PHA granules are often damaged by random and chain-end scission during solvent-based extraction processes. The final PHA purity and quality (e.g., molecular weight and polydispersity index) are thus affected by the extraction method. The solvent extraction method (especially halogenated solvents) provides the highest recovery with the highest purity and molecular weight (up to 98% and 0.8 MDa, respectively) (33, 35–38). However, because of the cost and the environmental impact of solvents, their use remains possible only at the laboratory scale, for research purposes. Beyond extraction, purification is also very important and has been found to greatly impact the processability of the polymer and its final properties (39); it was observed that multiple purification steps lead to better P(HB-co-HV) mechanical properties after processing. Because considerable quantities of hazardous solvents and energy are necessary during the extraction and purification steps, PHA production is not fully bio-benign. Moreover, the halogenated solvents used for the extraction can cause severe health issues in living organisms as well as environmental problems (40).

Above all, PHAs are biodegradable under various composting conditions (including home-compositing) and other natural environments (e.g., soil), making them particularly suitable to fight pollution by persistent plastics. As a result of successful tests on the biodegradation of PHA in activated sludge, compost, aqueous environments, and soil, the biodegradation of PHA blends with natural fillers in composting conditions is faster than with pure PHAs (41–43). This high biodegradation rate is the result of the involvement of a wide range of microorganisms and fungi, similar to the ones used for PHA biosynthesis, that can biodegrade PHAs in anaerobic and aerobic conditions (37, 44–48). Environmental factors such as temperature, pH, moisture, oxygen concentration, sunlight, the number/amount of microorganisms, as well as the chemical structure of PHA such as the percentage of 3 HV, are known to affect the rate of biodegradation of these polymers (49).

In summary, PHAs can be synthesized from agri-food wastes and residues using bioprocessed (microbial synthesis) and they are biodegradable under natural conditions and ambient temperature. They are thus promising candidates to turn the current linear plastic chain into a more sustainable cycle. Moreover, PHAs exhibit good usage benefits, with high gas/water barrier properties as well as good crystallization and mechanical properties; making them good candidates to prolong the shelf-life of food products. PHAs can also be converted into bioactive materials to increase their packaging usage benefit of reducing food losses.

## 3. The importance of bioactive functionality in food packaging

### 3.1. Bioactive packaging to better preserve food from deterioration

Commission Regulation [EC 450/2009 (50)] defined Active Packaging (AP) materials as follows “Materials and articles that are intended to extend the shelf-life, or to maintain or improve the condition, of packaged food; they are designed to deliberately incorporate components that would release or absorb substances into or from the packaged food or the environment surrounding the food.” As described in Table 2, various scavenging and releasing systems have been developed, aimed at decreasing the deterioration processes in food, such as microorganism growth, oxidation, etc. (61).

**Table 2:** The various scavenger and releasing systems developed for food applications (51, 52).

Type of system	Absorbed or released molecules	Potential uses of the active packaging	Shelf-life gain examples	References
Scavenger	Oxygen	Prevent aerobic microbial and mould spoilage, oxidation, rancidity, color change, vitamin loss	3–9 days (bakery products)	(53)
	Carbon dioxide	Absorb the excess CO_2_ produced by fermented and respired products	Up to 14 days (strawberries)	(54)
	Moisture	Prevent microbial and mold growth, maintain low humidity for dry and respired products	Up to 60 days (Portuguese cheese)	(55)
	Ethylene	Reduce ripeness and senescence for F&V	Up to 14 days (bananas)	(56)
Releasing	Carbon dioxide	Prevent microbial and mold growth	2 days (cod fillets)	(57)
	Ethanol		16 days (bread)	(58)
	Antimicrobial compounds		Up to 14–15 days (beef meat)	(59)
	Antioxidant compounds	Prevent lipid and vitamin oxidation	4 weeks (corn oil)	(60)

For example (62), showed that 0.5% of gallic acid in PHBV trays of meat, fish, or cheese packaging can induce the absorption of 7.7% of oxygen in 10 days (derived from food desorption or oxygen entrance through the packaging), thus limiting oxidation and microbial aerobic growth. Similarly, several authors have shown that the inclusion of different essential oils such as buriti oil (1.5 wt.%), orange leaf essential oil (2 wt.%), or thymol (6 or 8 wt.%) in chitosan, gelatin, or PLA films exhibited antimicrobial activity against *E. coli*, *P. aeruginosa*, *S. aureus*, and *B. subtilis*, as well as *C. albicans and A. niger,* making them suitable strategies to increase food shelf-lives in different sectors (63–66). Going beyond these studies demonstrating antimicrobial effects on only a single microbial species Alparslan et al. (67) tried to quantify the shelf-life gain obtained on fresh shrimp packed in gelatin film containing 2% orange leaf essential oil: the shelf-life increased from 8 days with gelatin film to 14 days with the active film. With the same objective of benefit quantification Suwanamornlert et al. (66) reported that the shelf-life of bread increased from 6 days (control with PLA packaging) to 9 days with PLA film containing 6 wt.% thymol.

It should be noted that essential oils extracted from natural aromatic plants (e.g., oregano, thyme, eucalyptus, rosemary, clove, cinnamon etc.) is often used as natural antioxidant, antimicrobial, and antifungal compounds for AP (68–70). Indeed, consumer demand for food products without synthetic chemical preservatives has promoted EO usage in the food packaging industry in recent years (68). As for all AP, the use of active components directly in the packaging material instead of in the food allow reduction of the use of preservatives, as the active component is slowly released toward the surface where it is needed (on the food surface).

**Figure 2:**
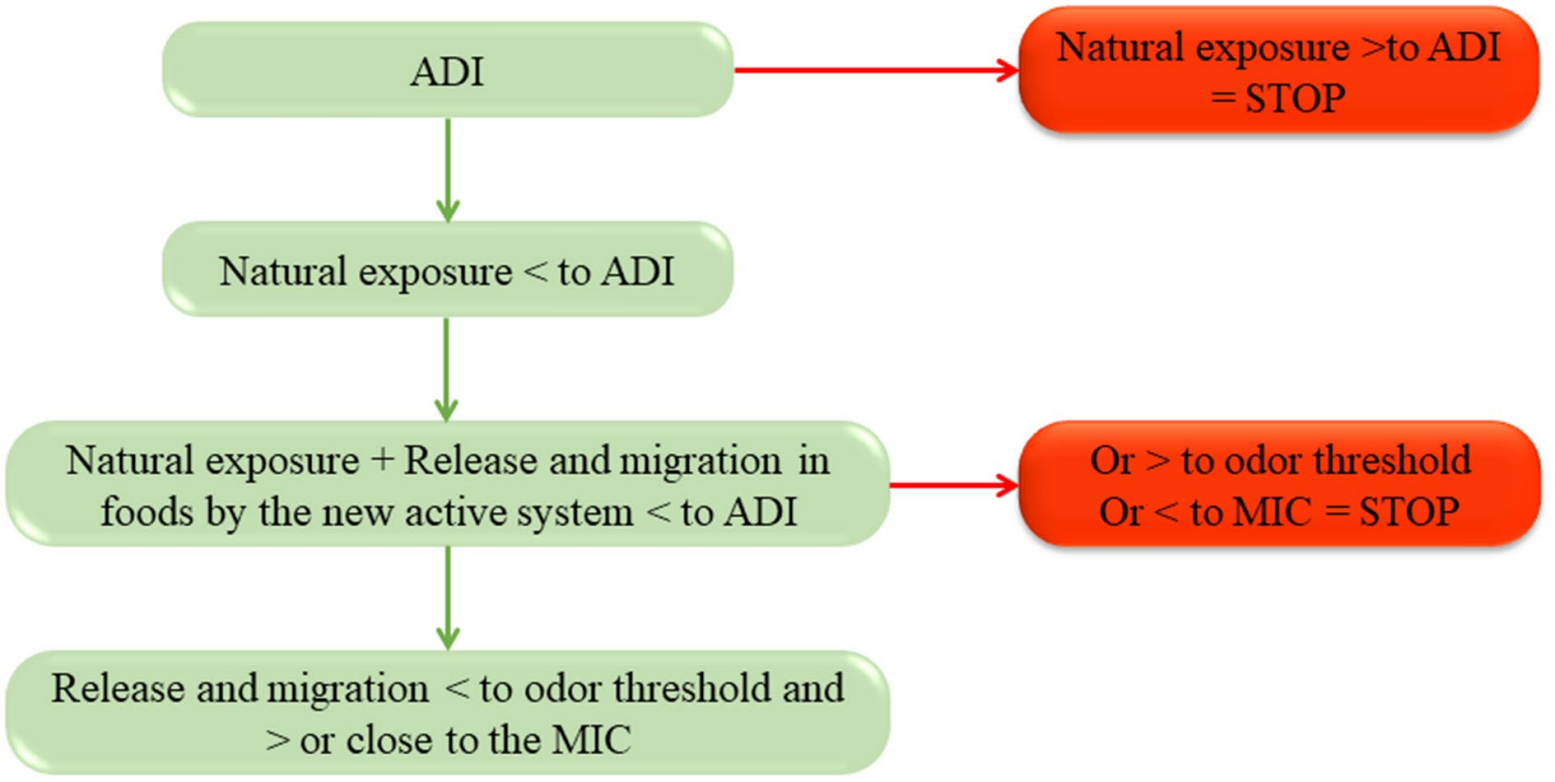
Principle of antimicrobial compound dimensioning.

To obtain safe and efficient active materials, the quantity of active compounds added in the formulation of active systems is defined by the expected efficiency but also regulatory needs and sensory aspects (e.g., active compounds that are also flavoring agents). Indeed, a specific admissible daily intake (ADI = the quantity of this specific compound that can be eaten without a negative impact on the health) is defined by regulations for all potential active substances (71). The use of the active molecules is possible only if the natural exposure of consumers to those substances from other (natural) sources (natural occurrence in food products due to their composition) does not exceed the ADI (Figure 2). The natural exposure depends on the country, food consumption habits, and the consumer’s age. This knowledge allows determination of the maximal quantity of the AC that can be used for active packaging (ADI minus the natural exposure) (Figure 2).

The choice of the best molecule to develop an active antimicrobial packaging also depends on the capacity of the molecules to act on the targeting of specific microorganisms. Consequently, the appropriate AC can differ from one product to another, depending on the microorganisms prone to growth on the product. To correctly design the active packaging, it is necessary to confirm that the quantity that can be used for the active packaging, while remaining below the difference between the ADI and natural exposure, is enough to reach the minimal inhibitory concentration (MIC) of the targeted microorganisms [EC 450/2009 (50)] (Figure 2). The use of modeling tools, taking into account the release and migration (for volatile molecules) or diffusion characteristics (for non-volatile molecules) of the AC in defined storage conditions (temperature, duration) of the product, as well as MIC for the targeted microorganisms allows precise determination of the quantities of antimicrobial agents to use in packaging to preserve the food product from deterioration (13, 72). For some molecules with high odor or taste, such as essential oils, the quantity of AC to use should be below the odor threshold and avoid organoleptic modifications (Figure 2), as stipulated in the European regulations [1935/2004 EC (73)] and [EC 450/2009 (50)].

### 3.2. Strategies for active functionality incorporation

**Figure 3:**
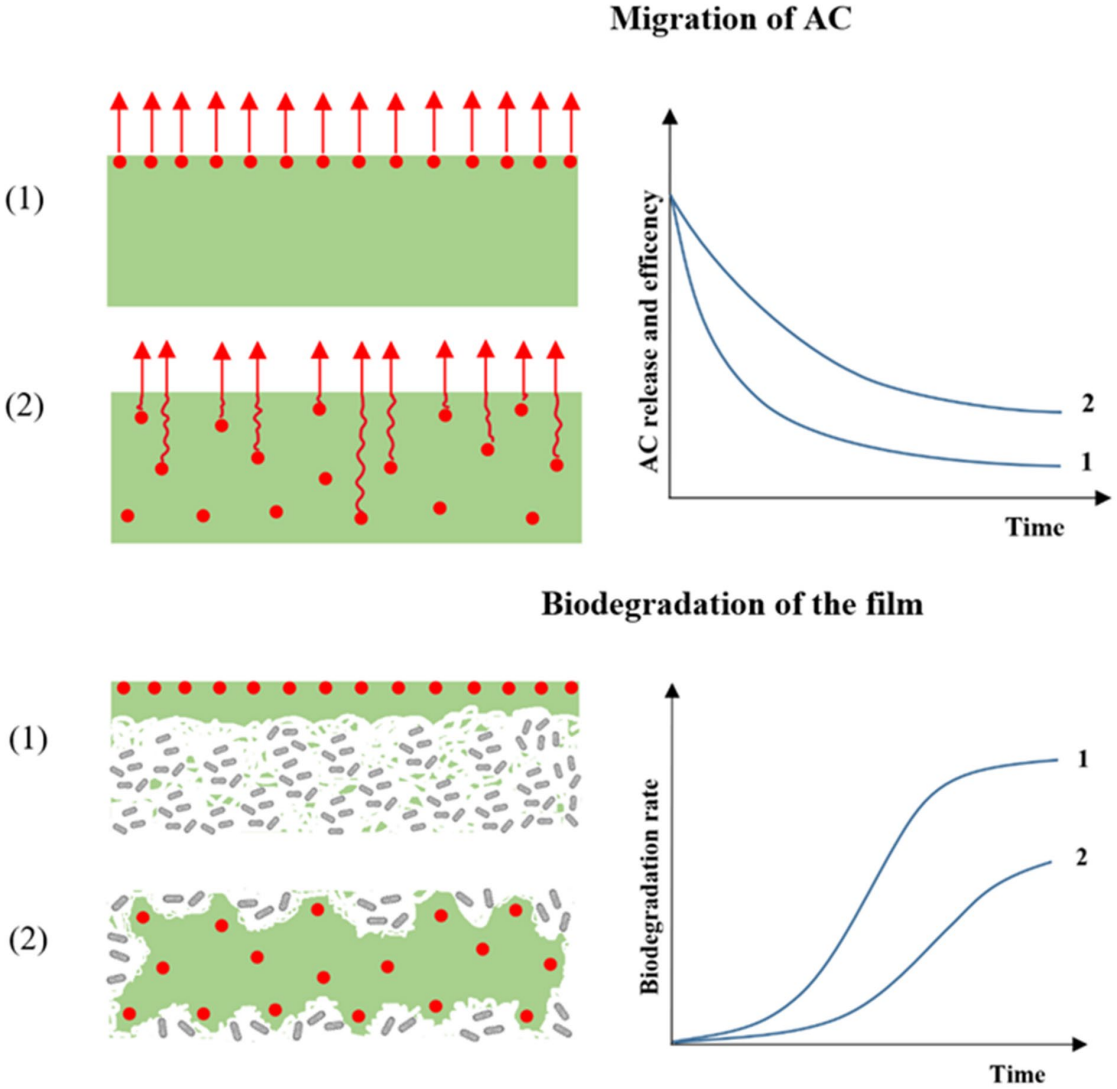
Hypothetical dependence of the active functionality incorporation methods, meaning (1) in coated and (2) “Throughout” material on the migration of AC and biodegradation of the film.

**Figure 4:**
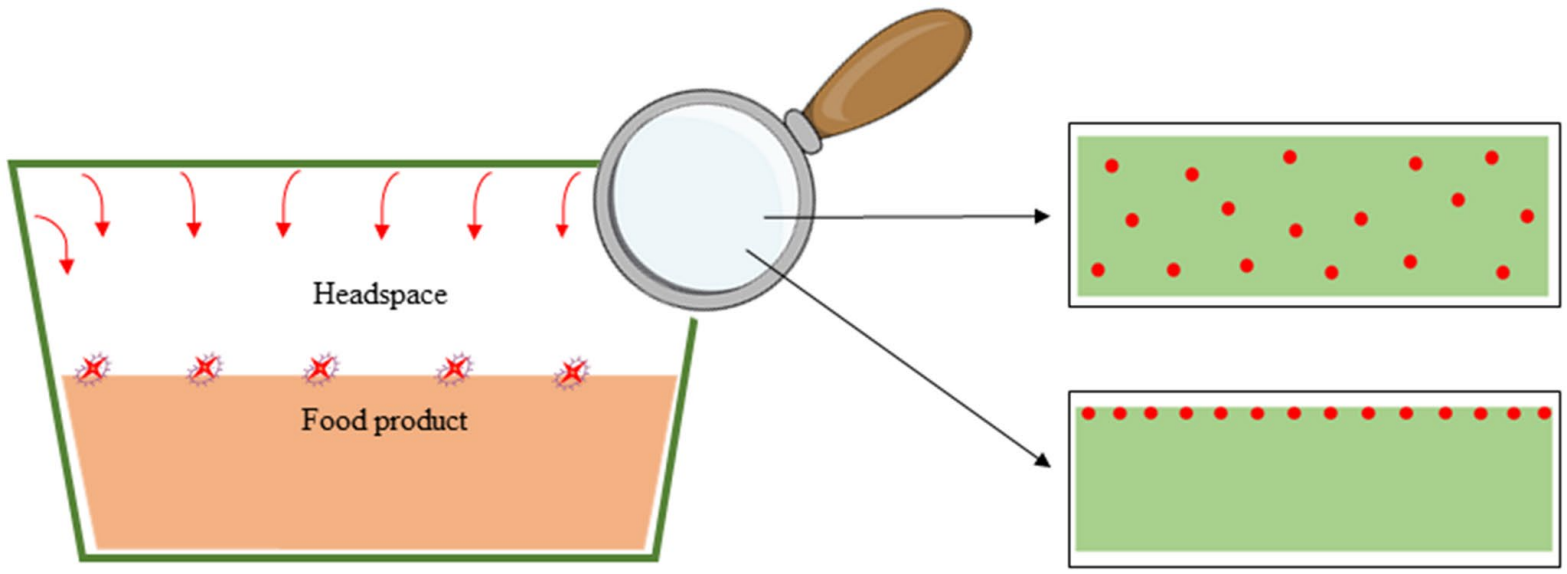
The various film structures and active volatile compound incorporation strategy (Active Compound Throughout and Active Compound in a surface coating) in packaging.

The impact of antimicrobial compounds, such as essential oil, on microorganism inhibition in food is dependent on the release and migration capacity of the molecules, and as a consequence on (1) the strategy of AC incorporation in the polymer material, (2) the volatility of the AC, and (3) the interactions and bonds between the component and the polymer material (74, 75). Indeed, different authors have shown that a film containing a homogenous concentration of AC throughout generated a slower and more sustained release than a film with an active coating concentrating the entire quantity of AC on the film surface (Figure 3) (76–78). This structure of AP and strategy of AC incorporation impact the preservative action of the AC on the food (Figure 4). For example, Wicochea-Rodríguez et al. (79) showed a four-fold lower release constant for carvacrol included in soy protein isolate film compared with the equivalent solution (carvacrol in soy protein isolate) coated on paper. Moreover, the authors also showed that the production of the same films with eugenol instead of carvacrol considerably increased the release of the molecule (release rate constant 100 times higher) due to the higher volatility of the molecule and lower retention by polymer, thereby inducing a different mechanism of release.

The choice to integrate AC throughout versus on the surface of polymers can also generate different degrees of loss of the molecules, depending on the production processing. Indeed, integration of AC directly in the polymer usually implies that the AC is subjected to thermal processes, such as extrusion or compression molding, meaning that highly sensitive molecules can be degraded, thus reducing their antimicrobial activity. By contrast, integration of AC on the surface of the polymer usually implies no thermal coating process, such as a bar, knife, spray coaters, or electrospinning, which preserves AC and its antimicrobial activity. The main principle of both of these physical incorporation methods is that the active components are physically contained or dispersed within the packaging material. These methods do not involve any chemical reactions or modifications of the active components, unlike sol–gel processes, surface immobilization, cross-linking, and covalent binding methods that involve chemical reactions (covalent and non-covalent bonds) between antimicrobial molecules and the film surface (77, 78, 80). The advantages and disadvantages of these incorporation methods are listed in Table 3.

**Table 3:** Advantages and disadvantages of physical and chemical active functionality incorporation methods.

Methods	Definition	Advantages	Disadvantages	References
Physical				
Extrusion	Continuous production of material by melting polymer and pushing it through the rotating screw	Low cost, high-volume production, suitable for different types of polymers, obtaining of a homogenous material	AC can be degraded due to the applied temperature in the process	(77)
Compression molding	Production of the film by applying pressure on pre-positioned polymer in the mold between hot plates			
Solution casting	Casting a polymer-dissolved solution on support where it solidifies thanks to the volatility of the solvent	Non-thermal process suitable for throughout formulations and coating	Use of solvents potentially toxic, difficulty to obtain a homogeneous material	(77, 81)
Knife coating	Spreading coating material on polymer support with a knife spreader	Simple, scalable, low cost	Use of solvents potentially toxic, flat surface requirement, difficulty to obtain a homogeneous material	(77)
Spray coating	Deposition of atomized droplets fluid as a coating on a polymer support			
Bar coating	Spreading coating material on a polymer support with a Mayer bar spreader	Simple, low cost	Limited scalability, use of solvents potentially toxic, flat surface requirement, difficulty to obtain an homogeneous material	
Dip coating	Immersion of the film substrate into a bath containing coating material followed by dwelling, withdrawal, and drying steps		Limited thickness control, use of solvents potentially toxic, difficulty to obtain a homogeneous material	
Layer by layer	Deposition of alternating layers of oppositely charged materials with wash steps in between	Low cost, suitable for large surface areas	Limited thickness control, a long processing time	(77, 82)
Spin coating	Application of coating materials on a flat support by using centrifugal force	Low cost, thin and uniform coating, quick drying	Not scalable, use of solvents potentially toxic, flat surface requirement	(77)
Electrospinning/spraying	Production of continuous nanoscale fibers/particles from polymer solution with the aid of a high-voltage electric field by pumping through a spinneret	Low cost, scalable, suitable for different types of polymers and AC	Use of solvents potentially toxic, difficulties in processing due to various impacts	(C (83).)
Chemical				
Sol–gel process	Formation of a gelatinous three-dimensional network (gel) from inorganic colloidal suspension (sol) (solution containing – antimicrobial and precursor of the inorganic phase) through hydrolysis and condensation	High-quality materials with homogeneity and purity	Long processing time	(77, 84)
Surface immobilization	Immobilization of AC on the film surface with materials that have compatible functional groups (proteins, enzymes, peptides, etc.)	Better stability and control of the AC release rate	Additional action could be needed to immobilize AC on the film surface (spacer, crosslinker); antimicrobial activity may be reduced due to the formed strong bonds.	(77)
Cross-linking	Formation of covalent bonds or ionic bonds to link the AC (with crosslinkers) to the polymer	Improvement of the thermal and mechanical stability of the material, modification of the release rate, and formation of a three-dimensional polymer network structure	Reduction of the bioactivity, increase the stiffness of the material, high cost	(85)

To overcome the issue of thermal degradation during thermomechanical shaping processes, attention has been given in the past two decades to different encapsulation strategies. Beyond mitigation and protection of AC from thermal degradation during packaging production, encapsulation may also help to control the release of the active molecules, especially if they are volatiles, and it is also used for that purpose (86). The term “encapsulation” was defined by Becerril et al. (87) and Stoleru and Brebu (88) as the technique that permits a substance (the active agent) to be encapsulated within another, resulting in tiny particles, the contents of which are gradually released under certain conditions. Hence, encapsulation strategies permit savings in regard to some active components as it is no longer necessary to compensate for the AC thermal degradation by spiking the initial load of AC in the material. In the same way, it allows minimization of the negative impact of the EOs on odors and organoleptic characteristics of the food products (89–91). Finally, encapsulation allows AC losses during storage (e.g., AC trapped into a polymer capsule) to be avoided and to trigger the release only when it is necessary, e.g., in food contact by action of humidity for instance as is the case for cyclodextrin-based capsules (92). Various carriers have been tested to develop encapsulation strategies, such as cyclodextrins, (nano)clays, nanofibers, and nanoparticles, all of which exhibit some advantages and disadvantages, as listed in Table 4 (87, 94).

**Table 4:** Advantages and disadvantages of the various encapsulation strategies used for active packaging.

Type of carriers	Definition	Advantages	Disadvantages	References
Cyclodextrins	The AC is trapped in cyclic oligosaccharides with a truncated cone shape, including an inner hydrophobic core and outer hydrophilic shell.	➔Highly effective control of the AC release, CDs are opened with high humidity ➔Suitable to encapsulate hydrophobic ACs ➔Stabilization of the volatile compounds	➔High price	(76, 92–94)
Nanoclays	The AC is trapped in a nanoparticle of mineral silicates (diameter size < 100 nm) with varying chemical compositions and morphologies (e.g., halloysite, montmorillonite, etc.).	➔Natural ➔Low price ➔Continuous release of the AC ➔Capacity to absorb volatile substances, such as off-flavors and off-odors	➔Migration capacity of clays: problematic for consumer safety	(95–97)
Nanofibers	The AC is trapped in fibers (1 nm < diameter size < 1 μm) produced by electrospinning techniques (use of an electric field on polymer solutions) and can be coated on a substrate or interlayers of biopolymer films.	➔Effective for trapping and release of the AC ➔High loading capacity ➔Low price	➔Difficult process because dependent on polymer and solvent characteristics, as well as processing parameters and environmental conditions, to obtain fibers with relevant morphologies (size, shape)	(83, 98, 99)
Biopolymeric carriers: nanospheres, nanocapsules	The AC is directly trapped inside a nanocarrier core encased in a thin polymer layer.	➔Biodegradable ➔Non-toxic ➔Low price ➔Controlled release of AC ➔Improved functional properties of the AP ➔Stabilization of the volatile compounds	➔Utilization of toxic solutions in production ➔Well suited to lab scale, but difficult to upscale	(100, 101)

## 4. How to reach the right balance of antimicrobial properties during the usage stage and biodegradation in the post-usage stage

To design bioactive and biodegradable packaging, it is crucial to consider the impact of natural antimicrobial components in polymers on the degradation capabilities of the entire packaging. Thus, it is necessary to achieve the right balance between the antimicrobial characteristics of packaging materials to increase the food shelf-life during the usage phase and the biodegradation of packaging during the post-usage phase. The influences of the incorporated bioactive components on the biodegradation properties of active biobased materials are reviewed in Table 5.

**Table 5:** Effects of bioactive components on the antimicrobial/antioxidant activity and biodegradation of biopolymers.

Polymer/Composite	Production method	Formulation strategy	Bioactive component	Antimicrobial/Antioxidant effect	Biodegradation condition	Biodegradation rate	AA or MP effect	Reference
						Delayed and/or slowed down		
Gliadin	Casting	Throughout	Cinnamaldehyde	-	Compost	Biodegradation rate decreased as the % of cinnamaldehyde increased	MP	(102)
Starch-PVA	Casting	Throughout	Neem EO, Oregano EO	-	Compost	EOs slightly decreased the biodegradation rate, especially Oregano EO	MP	(103)
Chitosan	Casting	Throughout	Buriti oil	*S. aureus, E. coli, P. aeruginosa*	Soil	Biodegradation rate decreased with increasing Buriti oil content, explained by increasing hydrophobicity with EO	MP	(63)
Starch	Casting	Throughout	Rosemary extract	*Antioxidant activity*	Compost	Biodegradation rate decreased by the presence of RE (time lag)	AA	(104)
Starch/Cassava/Sugarcane	Compression molding	Throughout	Oregano EO	*S. aureus, E. coli*	Soil	Biodegradation rate decreased with increasing Oregano EO %, explained by increasing hydrophobicity with EO	AA	(105)
Starch/PCL/Starch	Compression molding/Electrospinning	Throughout/Multilayer film	Carvacrol	*E. coli*	Compost	Slower biodegradation rate	AA	(106, 107)
PHBV/PLA-PHB	Electrospinning	Throughout/Bilayer film	Catechin	*Strong antioxidant activity*	Compost	Slightly slower disintegration rate	AA	(108)
Cassava/CMC	Casting	Coating	Turmeric	*A. niger*	Compost	15 μL TEO slowed the disintegration rate		(64)
PHB/PHA	Casting	Throughout	Grape seed lignin	*Antioxidant activity*	Compost	High % of lignin slowed the biodegradation rate	AA	(109)
Chitosan/Pigskin gelatin and Chitosan	Casting	Throughout	Boldo-do-Chile extract	*Psychrotrophic microorganisms, antioxidant activity*	Compost	Disintegration time reduced	MP	(110–112)
PHB	Casting	Throughout	Eugenol	*Salmonella sp*., *S. aureus*, *E. coli* and *A. niger*	Agricultural, sandy, and landfill soil	Biodegradation in sandy soil was slower than in other soil types		(113)
Pectin	Casting	Nanoemulsions/Throughout	Copaiba	*S. aureus, E. coli*	Soil	Slower biodegradation rate	AA	(114)
Chitosan	Casting	Throughout	Quercus extract	*B. subtilis, antioxidant activity*	Industrial compost, vineyard soil, and garden soil	QE reduced the biodegradation rate in all soil types	AA	(115)
Gelatin	Casting	Throughout	Citrus lignocellulosic fibers	*B. subtilis, S. aureus, E. coli, P. aeruginosa, C. albicans, strong antioxidant*	Soil	Increasing ratios of fibers decreased the biodegradation rate	MP	(116)
Chitosan/Cin/Chitosan	Casting	Coating/Multilayer film	Cinnamon	*S. aureus, E. coli*	Soil	Biodegradation rate of the films with 0.5% Cinnamon EO reduced	MP	(117)
PLA/PCL	Casting	Throughout	Green tea extract	*Antioxidant activity*	Compost	GTE with 30 wt.% decreased the biodegradation	MP	(118)
						Accelerated		
PLA-PHB	Compression molding	Throughout	D-Limonene	-	Compost	D-limonene accelerated the disintegration of the PLA-PHB-D-limonene blends	MP	(119)
PLA/Ag	Extrusion, injection molding	Throughout	Thymol	-	Compost	Thymol increased the biodegradation rate	MP	(120)
PLA/Cellulose Nanocrystals	Extrusion	Throughout	Lignin	*Xhanthomonas axonopodis* pv. *vesicatoria* and *Xhantomonas arboricola* pv. *pruni*	Compost	Increased biodegradation rate of the composites with 3 wt.% lignin		(121)
Starch	Casting	Throughout	Yerba mate extract	-	Compost	YME increased the biodegradation rate	MP	(122)
Bean starch	Casting	Throughout	Cocoa nibs extract	*Strong antioxidant*	Compost	CNE increased the biodegradation rate	MP	(123)
PCL	Extrusion	Throughout	Grape seed extract	*L. monocytogenes*	Soil	GSE increased the biodegradation rate	MP	(124)
PLA/Organoclay	Extrusion	Supercritical impregnation of the ACs in PLA/Organoclay	Thymol, Cinnamaldehyde	*S. aureus, E. coli*	Compost	Both thymol and cinnamaldehyde increased the biodegradation rate	MP	(125)
PLA	Casting	Throughout	Cocoa bean shells	*Strong antioxidant*	Water	CBS presence increased the biodegradation rate	MP	(126)
PLA	Casting	Throughout	Propolis	*E. coli, antioxidant activity*	Soil	Propolis addition increased the biodegradation rate		(127)
PLA/Nanoclay	Compression molding	Throughout/Nanoclay	Thymol	*S. aureus 8,325–4*, *E. coli*	Compost	Nanoclay increased the biodegradation rate	MP	(65)
PLA/Nanofibrillated cellulose	Casting	Throughout/Nanoclay	Thymol, Curry	Fungal growth	Soil	Thymol and NFC increased the biodegradation rate	MP	(128)
						Non affected		
Starch	Casting	Nanoemulsions/Throughout	Lemongrass EO	*S. aureus, E. coli*	Compost	LEO addition did not affect the biodegradation	-	(129)
Bacterial cellulose	Casting	Throughout	Lauric acid	*B. subtilis*	Compost	Biodegradation was not affected by LA addition	-	(130)
PLA/CNC/Chitosan	Extrusion, Electrospinning, Coating	Throughout/Multilayer film	Ethyl lauroyl arginate	*L. innocua, S. enterica*	Compost	LAE presence did not reduce the biodegradation rate	-	(131)

AA, antimicrobial activity; MP, modified properties.

All studies (Table 5) showed that the impact of active components on polymer biodegradation was due to either (1) the antimicrobial activities of the AC or (2) modification of polymer properties owing to the presence of AC (132, 133).

**Figure 5:**
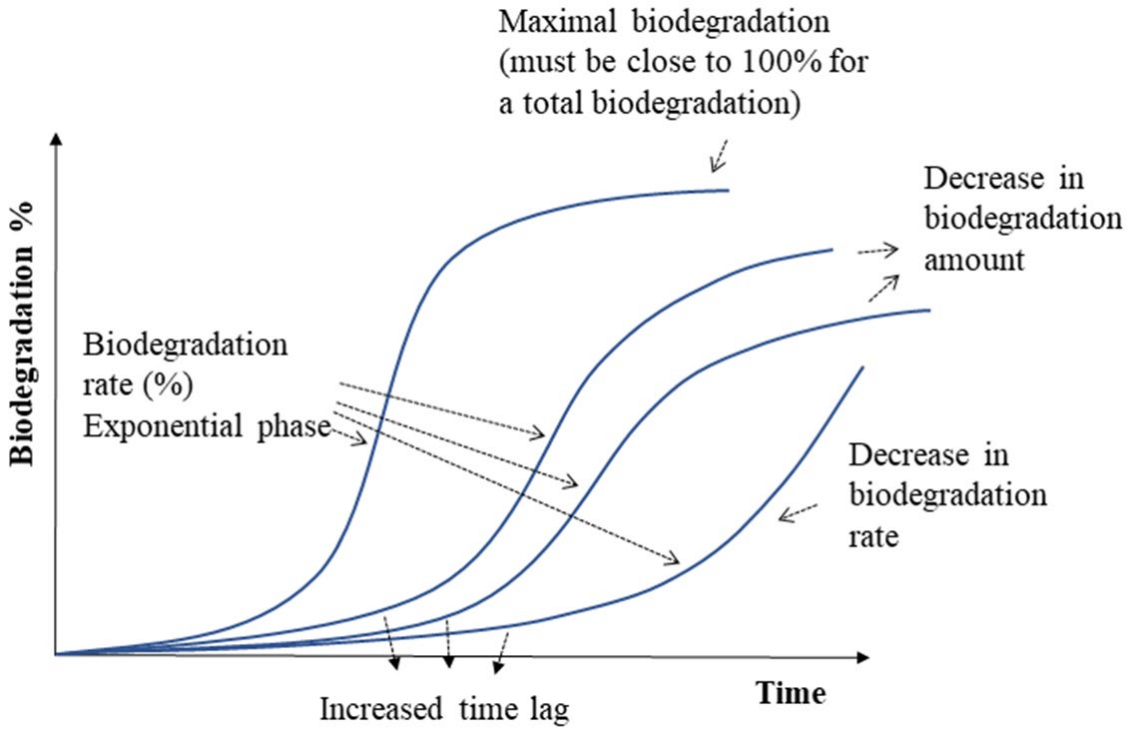
Different biodegradation scenarios for active biopolymers.

In the first case, essential oils and natural bioactive components have a high probability to hamper the biodegradation of biopolymers, as they are *per se* antimicrobial compounds and poorly biodegradable due to the presence of quaternary carbon atoms and fused or bridged ring systems (134). Most of these AC are extracted from thyme, mint, sage, parsley seed, and spearmint oil, and they are toxic to microorganisms, including those involved in biodegradation (135, 136). Various effects can be expected: a delay in the biodegradation kinetics (time lag) without further impact on the biodegradation rate, a slowing down of the biodegradation rate, or a decrease of the maximal biodegradation level, as shown in Figure 5. A combination of these three different impacts may also occur. An impact on the disintegration (the first step of the biodegradation process before assimilation by the microorganisms) has also been reported (108).

For instance, Tampau et al. (107), showed that biodegradation of a starch/PCL/starch multilayer containing carvacrol (15 wt.% PCL) in compost remained incomplete, with a maximum of 85%, after 45 days compared to the starch/PCL/starch film without carvacrol that is 100% biodegraded in the same conditions and duration, while Piñeros-Hernandez et al. (104) showed that biodegradation of starch film containing between 5 and 20% rosemary extract (polyphenol) was retarded in compost conditions (Table 5) but that the final biodegradation level was not affected.

However, although a decrease in biodegradation because of antimicrobial activity of AC was often observed, the majority of studies cover only active packaging with a non-encapsulation AC throughout the entity (Table 5). Consequently, the impact of the incorporation strategies, i.e., encapsulation or not, coating vs. incorporation throughout, etc. is very absent from the literature. Indeed, the presence of the AC on the surface of the packaging instead of throughout the entity increases the release of the molecules during the usage stage (see Section 3.2). Therefore, biodegradation should be impacted less, as the concentration of AC is lower and the polymer is more available for microorganisms at the post-usage stage (Figure 3). In this vein, Mustapha et al. (64), showed that a decrease in the coating thickness of starch with turmeric oil induced an increase in biodegradation. Consequently, a high level of importance should be placed on the design of active packaging to find the best formulation allowing the shelf-life to be increased without delaying the biodegradation of the material. For this, mathematical modeling could be developed, coupling the alteration of the microorganism’s growth in food and their impact on the product shelf-life, as well as the growth of the biodegradation microorganism and the impact on the end-of-life of the material.

In the second case, the addition of AC in polymers can either reduce or increase the polymer biodegradation due to modification of their properties. A decrease in biodegradation can be due to an increase in the molecular weight or hydrophobicity, or a decrease in flexibility, as has been observed with the presence of cinnamaldehyde (1.5 wt.% to 5 wt.%) in gliadin film during compost (102) or cinnamon (0.5 wt.%) in chitosan/starch film during biodegradation in soil (117). Indeed, EOs formed bonds with the polymer chains, and this cross-linking increased the glass transition temperature. It also restricted the entrance of water into the polymer matrix, which led to less swelling and lower water vapor diffusion coefficients. By contrast, the increase in biodegradation can be due to an increase in hydrophilicity, thermal stability, and chain mobility and a decrease in the molecular weight and crystallinity, as has been observed with the presence of green tea extract (from 10 to 30%) in PCL/PLA film in compost (118), or olive pomace (15%) in PHBV film during biodegradation in soil (137). It should be noted that AC encapsulated in nano clays, such as thymol in PLA-based nano-composites, cinnamaldehyde in PLA/organoclay, or curry/thymol in nanofibrillated cellulose/PLA, led to an increase in the film’s biodegradation owing to higher sensitivity to hydrolysis of the polymeric chains, which highlights the relevance of encapsulation of the AC (65, 125, 128). However, the impact of AC on the biodegradation of the polymer can differ due to the amount of the AC and reach a breaking point with an increase/or decrease of the AC quantity, whereby polymer biodegradation can decrease because of significant deterioration of the polymer properties. For example, the presence of 20 wt.% green tea extract in PCL/PLA accelerated the biodegradation of the entire material owing to increased hydrophilicity, but 30 wt.% green tea extract in PCL/PLA decreased the biodegradation of the entire material owing to an increase in crystallinity (118). By contrast, the integration of 3 wt.% lignin into a cellulose nanocrystals/PLA system resulted in acceleration of the disintegration of the film, but the presence of only 1 wt.% lignin in the system resulted in slower biodegradation due to a higher degree of crystallization of the material (121).

Finally, in some rare cases, the presence of AC in the polymer did not impact the biodegradation of the entire material, as observed with lemongrass EO and lauric acid in starch (129, 130), and ethyl lauroyl arginate (LAE) in a PLA/PLA-LAE/PLA-CNC/Chi structure (131).

Beyond AC incorporation that results in modification of the polymer properties, conditions met during biodegradation also affect the biodegradation rate (humidity, nature of the soil, etc.). Therefore, the type of active molecules, their incorporation strategy, their interaction with polymer, and on polymer properties, are among the many parameters that can affect (positively or negatively) the biodegradation of the bioactive material and should hence be taken into account. Beyond these observations, the comprehension of the interactions of these different parameters still needs to be deepened to better design active packaging, allowing for an increase in food shelf-life by maintaining or even enhancing the biodegradation of the packaging.

## 5. Conclusion

The continuous increase in plastic accumulation and production worldwide, and the very substantial environmental impact of food loss and waste, are the main reasons for improving the overall sustainability of food packaging materials. Therefore, an appropriate choice of material is paramount to tailoring sustainable food packaging. The use of materials derived from biobased sources, which do not compete with food and feed resources, has resulted in bioprocessing for environmentally friendly production. They are fully biodegradable by ensuring their digestibility under natural conditions, as is the case for PHAs, and they are showing great promise to enhance sustainability. Furthermore, bioactive materials help preserve food by minimizing deterioration, such as microbial development or oxidation, consequently reducing food loss and waste and its negative environmental impact. However, to design efficient antimicrobial packaging, the AC has to be present in sufficient quantity to reach the minimal inhibitory concentration of target microorganisms without surpassing the admissible daily intake. To achieve this goal, various strategies for the incorporation of AC in film (coating or throughout the polymer) have been developed with different encapsulation options, thereby enabling control of the release of the AC. However, it is crucial to achieve a proper equilibrium between antimicrobial functions during usage and biodegradability in the post-usage phase. Yet, in the case of natural active components (EOs, plant extracts, etc.), it has been shown that the amount and type of added AC, its incorporation strategy into the polymers, and its impact on the polymer’s properties can either positively or negatively impact the biodegradation properties of the biopolymer. Consequently, in the future, better comprehension and prediction of the impact of AC on the shelf-life of products and biodegradation of the material should be considered as a whole, through the use of modeling tools, to better design sustainable packaging.

## Author contributions

AR and FC wrote the first draft of the manuscript. All authors contributed to the conceptualization, design of the present review, manuscript revision, read, and approved the submitted version.

## Conflict of interest

The authors declare that the research was conducted in the absence of any commercial or financial relationships that could be construed as a potential conflict of interest.

## Publisher’s note

All claims expressed in this article are solely those of the authors and do not necessarily represent those of their affiliated organizations, or those of the publisher, the editors and the reviewers. Any product that may be evaluated in this article, or claim that may be made by its manufacturer, is not guaranteed or endorsed by the publisher.
